# Integrated Metabolomics and Network Pharmacology Investigation of Cardioprotective Effects of Myricetin after 1-Week High-Intensity Exercise

**DOI:** 10.3390/nu15061336

**Published:** 2023-03-09

**Authors:** Tianyou Li, Le Wang, Luting Wu, Yingquan Xie, Mengyun Chang, Dawei Wang, Long Yi, Xiaohui Zhu, Mantian Mi

**Affiliations:** 1 Research Center for Nutrition and Food Safety, Chongqing Key Laboratory of Nutrition and Food Safety, Institute of Military Preventive Medicine, Army Medical University, Chongqing 400038, China; 2 Chongqing Medical Nutrition Research Center, Chongqing 400038, China

**Keywords:** high-intensity exercise, myricetin, metabolomics, network pharmacology, molecular mechanism

## Abstract

Cardiovascular adverse effects caused by high-intensity exercise (HIE) have become a public health problem of widespread concern. The therapeutic effect and metabolic regulation mechanism of myricetin, a phytochemical with potential therapeutic effects, have rarely been studied. In this study, we established mice models of different doses of myricetin intervention with 1 week of HIE after intervention. Cardiac function tests, serology, and pathological examinations were used to evaluate the protective effect of myricetin on the myocardium. The possible therapeutic targets of myricetin were obtained using an integrated analysis of metabolomics and network pharmacology and verified using molecular docking and RT-qPCR experiments. Different concentrations of myricetin improved cardiac function, significantly reduced the levels of myocardial injury markers, alleviated myocardial ultrastructural damage, reduced the area of ischemia/hypoxia, and increased the content of CX43. We obtained the potential targets and regulated metabolic network of myricetin by combined network pharmacology and metabolomics analysis and validated them by molecular docking and RT-qPCR. In conclusion, our findings suggest that myricetin exerts anti-cardiac injury effects of HIE through the downregulation of PTGS2 and MAOB and the upregulation of MAP2K1 and EGFR while regulating the complicated myocardial metabolic network.

## 1. Introduction

The metabolic equivalent (MET) is an international commonly used index for evaluating exercise intensity. MET usually depends on the ratio of oxygen consumption per kilogram of body weight under different exercise conditions compared to that at rest. The World Health Organization guidelines note that exercise intensity ≥ 6 MET is considered HIE and is common in people’s daily lives [1]. Although moderate exercise to promote cardiovascular health is commonly accepted, studies have found that the ratio of cardiovascular disease and the increase in MET have a U-shaped relationship that first decreases and then increases [2]. A population study found that the primary cause of sudden death among NCAA (National Collegiate Athletic Association) basketball and football athletes who often engage in intensive training and competition is sudden cardiac death, and the rate of sudden death is significantly higher than that among college students of the same age [3]. Military personnel who have completed many years of service have a higher cardiovascular risk in midlife than their non-serving peers [4]. Studies have found that continuous HIE may increase serum myocardial injury markers, reduce cardiac function, and produce abnormal electrophysiological changes [5,6]. Related mechanisms may include changes in metabolic condition, inflammation, and oxidative stress damage [7,8]. Some cardioprotective drugs commonly used in clinical practice have shown the potential to alleviate myocardial injury after HIE in animal experiments [9]. However, owing to the disadvantages of the “one-drug, one-target” theory, and the side effects of chemotherapeutic drugs [10], these have not been applied in practice for protection in HIE.

In recent years, flavonoids have attracted widespread attention owing to their high safety and many benefits to human health [11]. Among them, myricetin (3,5,7,3′,4′,5′-hexahydroxyflavonol, MYR), which is widely found in berries, vegetables, and red wine, has various biological functions [12]. In terms of cardiovascular effects, studies have reported that myricetin can alleviate harmful oxidative stress by regulating the antioxidant enzyme system [13,14]. Inflammatory responses can also be attenuated by interfering with the NF-κB pathway [15]. In addition to the aforementioned mechanisms [16,17,18], myricetin can also reduce the progression of coronary atherosclerosis by regulating the immune status of CD36 macrophages [19]. However, there are few studies on the role of myricetin in HIE, and the related mechanisms and targets have not been fully elucidated.

To investigate the protective effect of myricetin in HIE and its metabolic regulation mechanism, we established an animal model in which different doses of myricetin were administered via gavage for 4 weeks. Mice then performed HIE continuously for 1 week. The degree of myocardial injury was assessed in serological testing, cardiac function measurement, and pathological observation. Integrated analysis using metabolomics and network pharmacology combined with molecular docking and RT-qPCR was used to reveal the potential interfered targets and myocardial metabolic network. We found that myricetin can significantly alleviate myocardial injury caused by HIE. This mechanism might be related to the regulation of the myocardial metabolic network. These findings from the animal model may help deepen the understanding of the pharmacological effects of myricetin and provide theoretical support for the discovery of new strategies for protection in HIE.

## 2. Materials and Methods

### 2.1. Databases and Software

The databases and software used in this study are shown in Table 1 and Table 2, respectively.

### 2.2. Animal Model Establishment and Sample Collection

Forty SPF male C57BL/6 mice, body weight 20 ± 2 g, were obtained from SJA Laboratory Animal Co., Ltd. (Changsha, China). This animal experiment protocol was approved by the Ethics Committee of the Army Medical University (No. AMUWEC20202155). Animals were housed in the SPF Experimental Animal Center of the Army Medical University. The ambient temperature was 21–23 °C, the humidity was 40–60%, and mice maintenance food (1025, HFK bioscience, Beijing, China) and sterile water without additives were provided ad libitum. The food ingredients are shown in Supplemental Table S1.

The experimental animals were randomly divided into four groups (*n* = 10): the sedentary group (sed), high-intensity exercise group (ex), low-dose myricetin gavage group (100 myr), and high-dose myricetin gavage group (200 myr). The 100 myr and 200 myr groups received 100 mg/kg/d and 200 mg/kg/d doses of myricetin (purity ≥ 98%, Must bio-technology, Chengdu, China) dissolved in normal saline for gavage; the other two groups received normal saline in gavage. The intervention period lasted for 4 weeks. After the gavage intervention, mice performed 7 days of HIE training. The training protocol is exhausting swimming at the same time per day in a week. Exhaustive criteria were failure to reach the surface of the water for 5 s and blunted righting reflex. All mice that completed the training protocol were killed with isoflurane anesthesia. The whole blood was left to stand at room temperature for 30 min, centrifuged at 1500× *g* in a 4 °C centrifuge for 15 min to obtain serum, and stored in a −80 °C refrigerator. Part of the left ventricular tissue was fixed in a 4% paraformaldehyde solution at room temperature for histological experiments. Another part of the tissue was fixed in a 2.5% glutaraldehyde solution at 4 °C for transmission electron microscopy, and the remaining heart tissue was stored in a −80 °C refrigerator for molecular biology experiments.

### 2.3. Echocardiography Analysis

Mice that completed the HIE training were continuously anesthetized with 3% isoflurane. We used B-mode ultrasound in a cardiac color Doppler ultrasound instrument (vivid E9, General Electric Company, Boston, MA, USA) and a left-ventricular long-axis view; we then switched to M-mode ultrasound mode to measure the left ventricular anterior wall thickness, left ventricular inner diameter, and left ventricular posterior wall thickness, and calculated the ejection fraction (EF%) and fractional shortening (FS%). Three mice were randomly selected from each group for testing. The echocardiography operator was blinded to the group allocation during the experiment.

### 2.4. Serological Analysis and ELISA

Aspartate aminotransferase (AST), lactate dehydrogenase (LDH), creatine kinase (CK), and creatine kinase isoenzyme (CK-MB) were measured using an automatic biochemical analyzer (3110, Hitachi, Tokyo, Japan) and supporting biochemical kits. Troponin I was determined using an enzyme-linked immunosorbent assay kit (ELISA) (E-EL-M1203c, Elabscience, Wuhan, China), in strict accordance with the instructions of the kit.

### 2.5. Hematoxylin and Eosin (HE) Staining

Myocardial tissues were fixed in a 4% paraformaldehyde solution, dehydrated, embedded, and routinely sectioned for pathology. The obtained paraffin sections were dewaxed and rehydrated, and then stained using an HE staining kit (G1120, Solarbio, Beijing, China). The experimental process was performed in strict accordance with the instructions of the kit. After staining, a neutral resin was used to seal the slides. Microscopic examination and photography were performed using an optical microscope (BX51, Nikon, Tokyo, Japan).

### 2.6. Transmission Electron Microscopy

Fixed myocardial tissue was immersed in osmic acid for further fixation, dehydrated with a gradient ethanol solution, and embedded with Epon812. Double staining was conducted with uranyl acetate and lead citrate. After making ultrathin sections, the samples were observed under a transmission electron microscope (JEM-1400, JEOL, Tokyo, Japan). Quantitative statistics were performed on the damage ratio of mitochondria (visual fields *n* = 10 per group).

### 2.7. Hematoxylin-Basic Fuchsin-Picric Acid (HBFP) Staining

Paraffin sections were dewaxed to water, dipped in hematoxylin solution for 30 s, rinsed with tap water for 1 min, differentiated with a hydrochloric acid solution for 3 s, washed with tap water, blue-stained with ammonia water for 1 min, and washed with tap water. The sections were then dipped into fuchsin basic solution for 3 min, washed with tap water, and dipped in pure acetone solution for 8 s. Sections were repeatedly dipped in picric acid acetone solution until the red color disappeared. The slides were then sealed using a neutral resin. Three mice were randomly selected in each group; three 400× visual fields were randomly selected to be photographed, and the IOD of positive areas was calculated using ImageJ software.

### 2.8. Immunofluorescence Staining

Paraffin sections were dewaxed in water, immersed in sodium citrate solution, and heated in a microwave oven for antigen retrieval. Antigen blocking was performed after antigen retrieval with Immunol Staining Blocking Buffer (P0260, Beyotime, Shanghai, China), and sections were then incubated overnight with CX43 antibody (1:100 dilution, CST, Boston, MA, USA) at 4 °C. The next day, the sections were briefly washed with phosphate-buffered saline containing Tween 20 and incubated with Alexa Fluor 488-conjugated secondary antibody. Finally, the nuclei were stained and sealed with a DAPI-containing kit (P0131, Beyotime, Shanghai, China). Three mice were selected in each group; three randomly selected 400× view fields were taken using a fluorescence microscope (BX51, Nikon, Tokyo, Japan) for examination and photography, and the average fluorescence density was analyzed using ImageJ software.

### 2.9. Bioinformatics Analysis for Metabolomics

The raw metabolomics data originated from the Metabolight database (ID MTBLS5608). The raw data were transformed in file format to MzXML using ProteoWizard, and XCMS software was used for peak alignment, retention time correction, and peak area extraction. SIMCA 16 was used for Principal Components Analysis and Orthogonal Partial Least Squares-Discriminant Analysis, and the R language clusterProfiler package was used for cluster analysis and KEGG enrichment analysis. The identification criteria of differential metabolites were *p* < 0.05, Fold Change > 1.5 or Fold Change < 0.67, and Variable Importance in the Projection value of OPLS-DA analysis > 1.

### 2.10. Network Pharmacology Analysis

Potential interference targets for myricetin were obtained from the SwissTarget prediction database. Gene Ontology analysis (GO), pathway enrichment analysis (KEGG), and MCODE analysis of interference targets were implemented using the Metascape database. Protein-protein interaction (PPI) analysis was implemented using the String database. The phytochemical-metabolic pathway-metabolite-interference target network diagram was created using Cytoscape.

### 2.11. Molecular Docking

The crystal structure of the target protein was acquired from the Uniprot database, and the 3D structure of myricetin was acquired from the PubChem database. Chem3D was used to minimize the energy of myricetin under the MMFF94 force field. The acceptor proteins were treated with PyMol to remove unnecessary small molecules or solvent molecules. The docking box was then set up for the entire protein. In addition, ADFRsuite was used to convert small molecules and receptor proteins into the pdbqt format. AutoDock Vina was used for molecular docking. The docking parameters maintained the default settings. The docking results with the highest scores were visualized using PyMol.

### 2.12. Quantitative Real-Time PCR

RNAiso (9109, Takara, Tokyo, Japan) was used to extract total RNA from myocardial tissue. The RNA concentration and quality were detected using a spectrophotometer (Nanodrop 2000, Thermo Scientific, Waltham, MA, USA), and the mRNA was reversed to cDNA using PrimeScript RT Master Mix (RR036A, Takara, Japan). Quantitative real-time PCR was then performed using TB Green^®^ Premix Ex Taq™ II (RR820A, Takara, Tokyo, Japan), with a QuantStudio™ 3 system (A28137, Thermo, Waltham, MA, USA). The expression levels of target genes were normalized to Actb. The primer sequences for the target genes are listed in Table 3.

### 2.13. Statistical Analysis

Data are expressed as mean ± standard deviation (SD). Graphpad 8 was used for statistical analysis. One-way analysis of variance was used for comparisons of multiple groups. The LSD *t*-test was used to make intergroup comparisons followed by Bonferroni post hoc tests to control for multiple comparisons. *p* < 0.05 was considered statistically significant.

## 3. Results

### 3.1. Myricetin Improved Cardiac Function and Reduced Myocardial Injury Marker Levels

**Figure 1:**
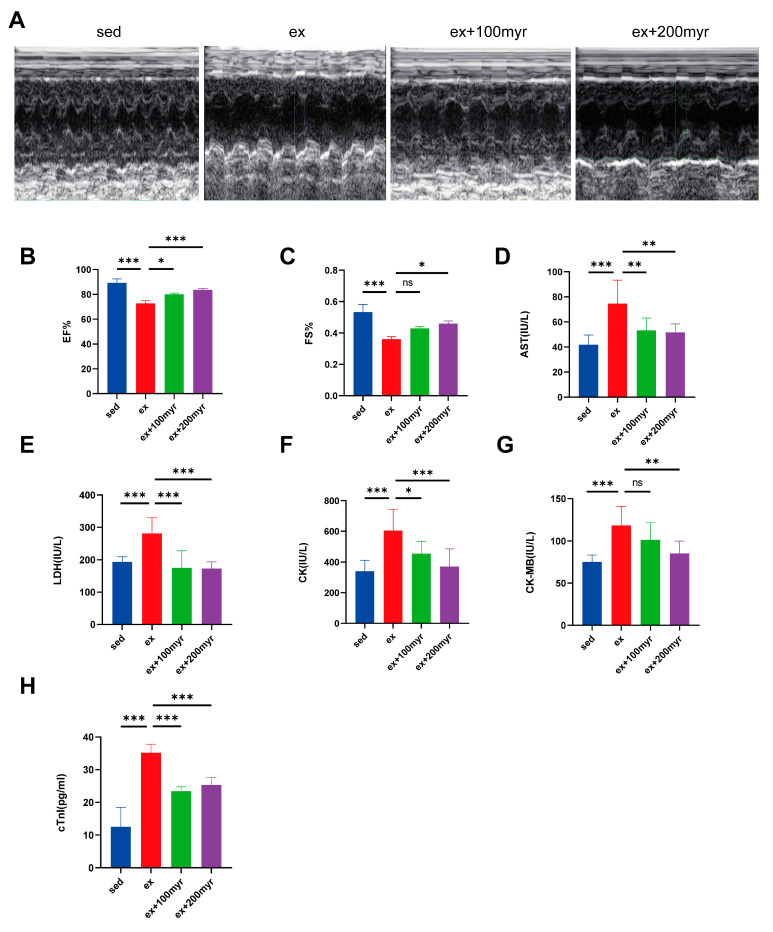
Effects of myricetin on cardiac function and serum marker levels in HIE. (**A**) Representative M-mode echocardiograms; (**B**) ejection fraction (EF%) values; (**C**) left-ventricular short-axis shortening (FS%) values; (**D**) AST; (**E**) LDH; (**F**) CK; (**G**) CK-MB; and (**H**) mouse serum troponin I (cTnI). The data are presented as mean ± SD. * *p* < 0.05, ** *p* < 0.01, *** *p* < 0.001, and ns represents no statistical difference.

Representative echocardiograms of the different groups are shown in Figure 1A. Both EF% and FS% decreased significantly in the HIE group; the myricetin intervention enabled significant improvement in cardiac function in the 200 myr group (Figure 1B,C). All serum cardiac injury marker levels were significantly higher in the HIE group than in the sed group. AST, LDH, CK, CK-MB, and mouse serum troponin I decreased significantly in the group that received two doses of myricetin compared with the HIE group, except for the decrease in CK-MB level in the 100 myr group, which was not statistically significant (Figure 1D–H).

### 3.2. Myricetin Alleviated Myocardial Pathological Changes Induced by HIE

**Figure 2:**
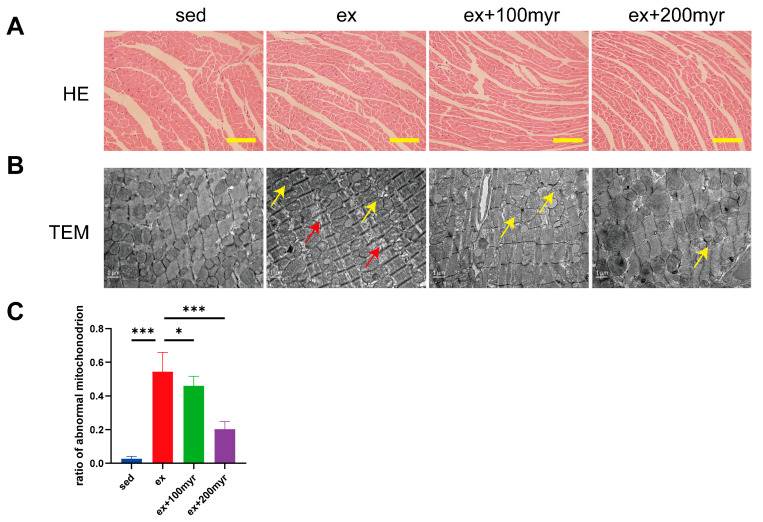
Effect of myricetin on myocardial pathology. (**A**) Representative HE staining results. Scale bar in the figure is 100 μm; (**B**) representative transmission electron microscopy results; scale bar in the figure is 1 nm, magnification 15,000×; and (**C**) proportion of abnormal mitochondria. Yellow arrows indicate sparse arrangement of myocardial fibers, and red arrows indicate swollen and dissolved mitochondrial cristae. The data are presented as mean ± SD. * *p* < 0.05; *** *p* < 0.001.

HE staining suggested no obvious pathological change in all groups (Figure 2A). However, significant pathological changes in ultrastructure were observed in transmission electron microscopy. The ex group revealed a sparse arrangement of myocardial fibers (yellow arrows) and swollen and dissolved mitochondrial cristae (red arrows). Different concentrations of myricetin intervention could alleviate the ultrastructure damage of myocardial fibers (Figure 2B). The percentage of abnormal mitochondria also decreased significantly with increasing myricetin concentrations (Figure 2C).

### 3.3. Myricetin Decreased Myocardial Ischemia and Hypoxia Areas and Restored CX43 Content after HIE

**Figure 3:**
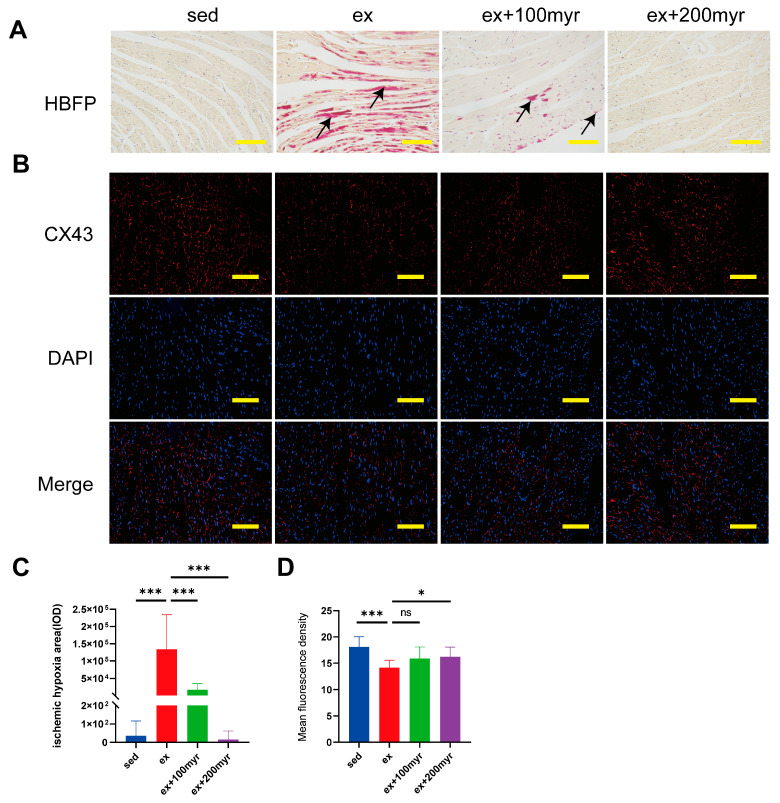
Effect of myricetin on the myocardial ischemic-hypoxic area and CX43 protein content. (**A**) Representative hematoxylin basic magenta picrate (HBFP) staining results. The red-stained area is the ischemic-hypoxic myocardium, and the yellow area is the normal myocardium; (**B**) representative immunofluorescence staining results. CX43 shows red fluorescence, and the nucleus shows blue fluorescence; (**C**) myocardial ischemic-hypoxic area; and (**D**) CX43 mean fluorescence density. Black arrows indicate ischemic-hypoxic myocardium. The data are presented as mean ± SD. * *p* < 0.05, *** *p* < 0.001, and ns represents no statistical difference; scale bar = 100 μm.

The red-stained area of HBFP staining (black arrow) is the ischemic-hypoxic myocardium (Figure 3A). The myocardial gap junction CX43 showed red fluorescence after staining, and the nuclei showed blue fluorescence (Figure 3B). In quantitative statistics, we found that the area of ischemic-hypoxic myocardium was significantly higher in the ex group than in the sed group. The area of ischemia-hypoxia was significantly reduced in the different myricetin groups (Figure 3C). The mean fluorescence density of CX43 was significantly lower in the ex group than in the sed group, with no significant increase in the 100 myr group and a significant increase in the mean fluorescence density in the 200 myr group compared with the ex group (Figure 3D).

### 3.4. Dramatic Changes in Myocardial Metabolism after HIE

**Figure 4:**
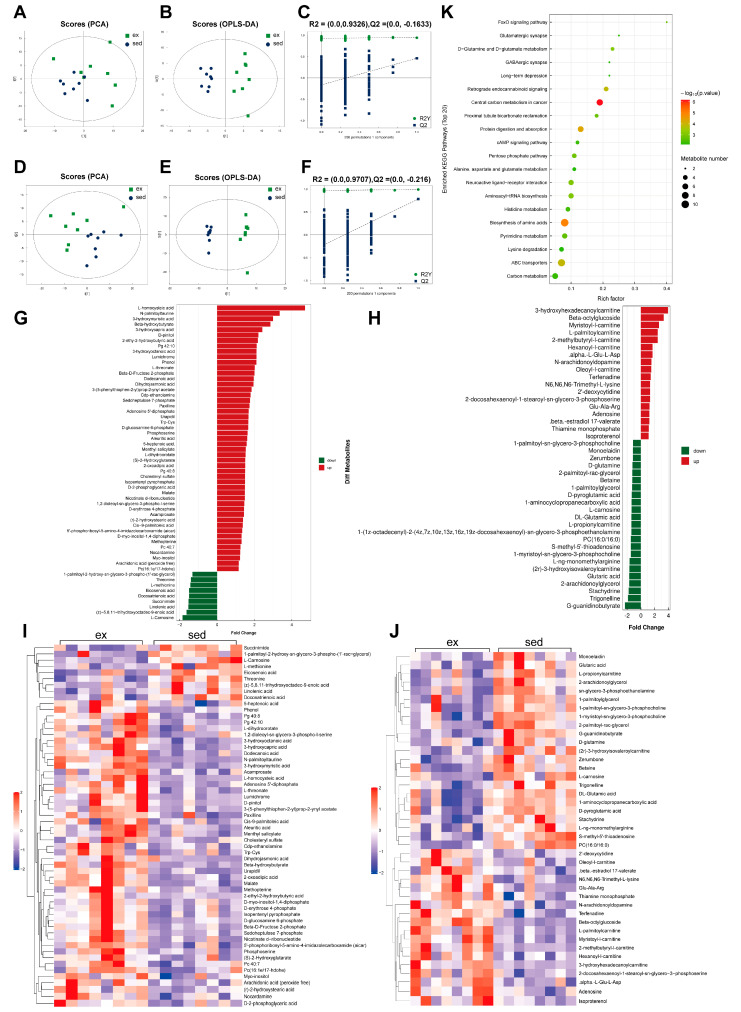
Effects of HIE on myocardial metabolism. (**A**) Positive-ion mode PCA score plot; (**B**) positive-ion mode OPLS-DA score plot; (**C**) positive-ion mode OPLS-DA 200-permutation test score plot; (**D**) negative-ion mode PCA score graph; (**E**) negative-ion mode OPLS-DA score plot; (**F**) negative-ion mode OPLS-DA 200-permutation test score plot; (**G**) butterfly plot of the expression fold change in negative-ion mode metabolites; (**H**) butterfly plot of the expression fold change in positive-ion mode metabolites; (**I**) heat map of negative-ion mode metabolites; (**J**) heat map of positive-ion mode metabolites; and (**K**) bubble map of top 20 metabolic-enriched pathways.

The positive ion mode of PCA analysis in sedentary versus HIE status revealed significant separation between samples of the two statuses (Figure 4A). The supervised model OPLS-DA (R2Y = 0.941, Q2 = 0.461) and 200-times-permutation validation analysis further confirmed a significant difference in metabolism between the two statuses (Figure 4B,C). In negative-ion mode, the separation of the two statuses in the PCA plot and main quality parameters (R2Y = 0.991, Q2 = 0.781) from the OPLS-DA model showed that the two statuses were effectively distinguished from each other. These results were verified by 200-times-permutation validation analysis (Figure 4D–F). The expression changes in the differential metabolites in the two ion modes are shown in butterfly diagrams (Figure 4G,H). The inflammation-related metabolite arachidonic acid increased after HIE, and the anti-oxidative stress-related metabolites L-carnosine, DL-glutamic acid, and D-glutamine decreased after HIE. The correlation heat map of differential metabolites depicts the distinct metabolite change patterns between sedentary and HIE (Figure 4I,J). The KEGG enrichment analysis revealed the top 20 enriched pathways (Figure 4K). Glucose metabolism, amino acid metabolism, and anti-oxidative stress-related pathways were enriched.

### 3.5. Network Pharmacology Analysis of Myricetin

**Figure 5:**
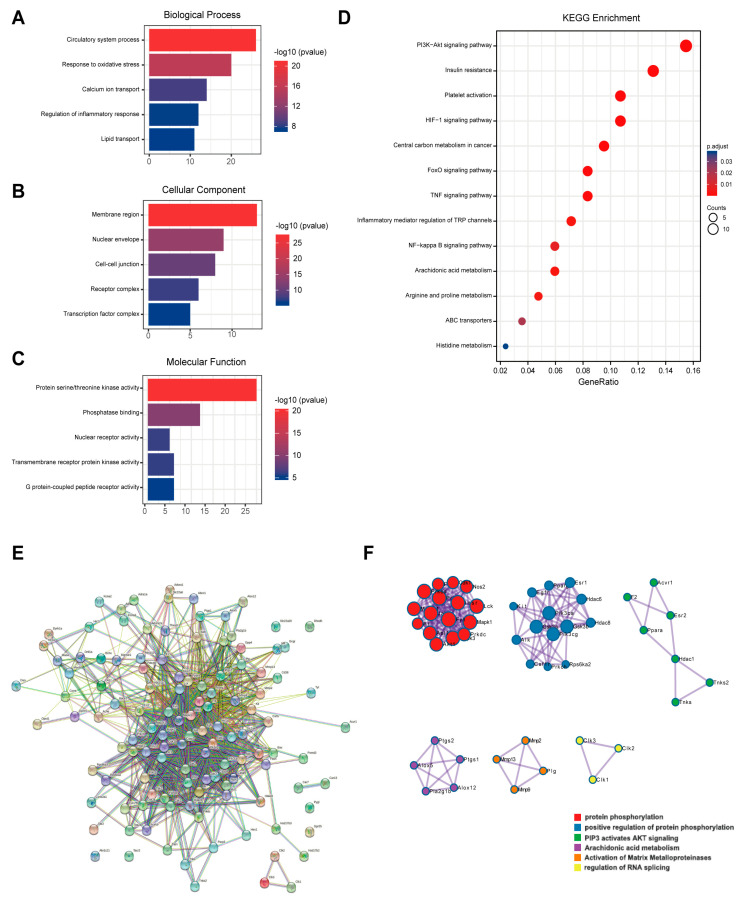
Results of network pharmacological analysis. (**A**) Biological process of GO analysis; (**B**) cellular component of GO analysis; (**C**) molecular function of GO analysis; (**D**) KEGG pathway enrichment; (**E**) network diagram of PPI analysis; and (**F**) results of MCODE analysis.

A total of 98 possible action targets of myricetin were found in the SwissTarget prediction database. The detailed gene names are shown in Supplemental Table S2. Predicted targets were analyzed using GO. The high-enrichment biological processes related to myocardial activity were circulatory system processes, oxidative stress response, calcium ion transfer, inflammatory response, and lipid transfer (Figure 5A). Furthermore, cellular components were membrane regions, nuclear periplasm cellular junctions, receptor complexes, and transcription factor complexes (Figure 5B), and molecular functions were protein-serine-threonine kinase activity, phosphatase binding, nuclear receptor activity, transmembrane protein receptor kinase activity, and G-protein-coupled peptide receptor activity (Figure 5C). The KEGG enrichment analysis revealed that the enriched pathways were mostly focused on glycan, amino acids, antioxidants, and anti-inflammatory-related pathways (Figure 5D). PPI analysis showed that there were 98 points and 594 edges in the protein interactions network map, and the degree of interaction enrichment was *p* < 1 × 10^−16^ (Figure 5E). MCODE analysis was used to screen the hub targets in the PPI network as well as the functions. The results are shown in Figure 5F. Protein phosphorylation-related processes, arachidonic acid metabolism, and the PIP3-AKT pathway may be the key pharmacological effects of myricetin.

### 3.6. Integrated Analysis of Metabolomics and Network Pharmacology

**Figure 6:**
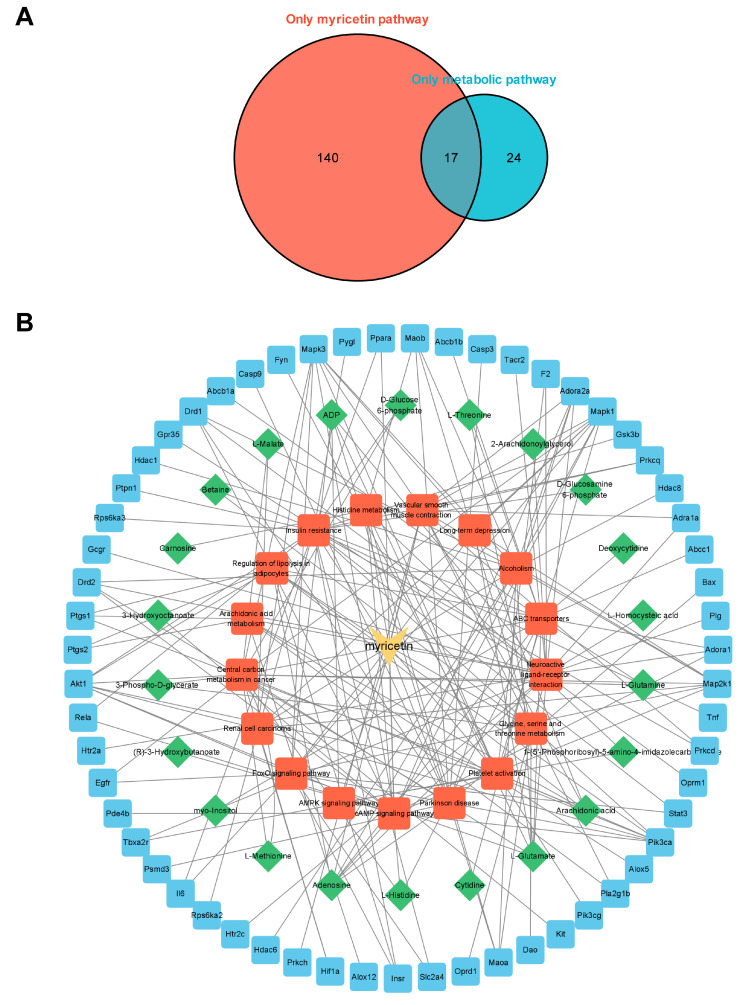
Network pharmacological analysis. (**A**) Venn plot of the relationship of metabolomics and network pharmacology enrichment pathways. Numbers in the red area indicate metabolic pathways enriched only in targets of myricetin, and numbers in the blue area indicate metabolic pathways enriched only in high-intensity exercise. Overlapping area indicate a common metabolic pathway. (**B**) Myricetin-pathways-metabolites-targets networks of key metabolites and targets.

To comprehensively study the regulation of myocardial metabolic networks according to myricetin, we established a network diagram of myricetin-metabolic pathways-metabolites-targets based on the common metabolic pathways discovered using metabolomics and network pharmacology. There were 17 common pathways (Figure 6A). The names of the pathways mentioned above are shown in Supplemental Table S3. Cytoscape was used to visualize the myricetin-common metabolic pathway-differential metabolites-possible therapeutic target network relationships (Figure 6B). The above results reflect the complex metabolic regulation of myricetin.

### 3.7. Molecular Docking

**Figure 7:**
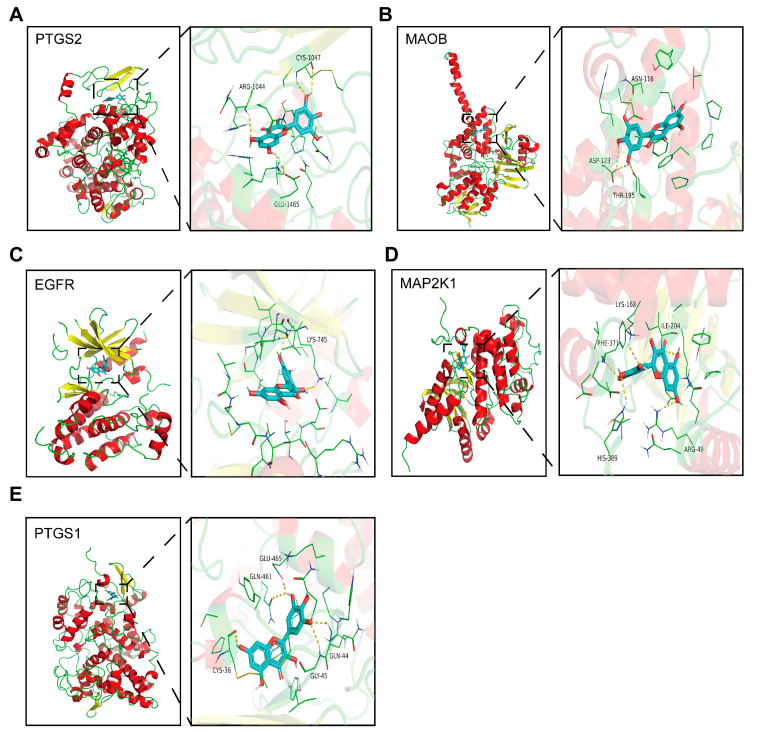
Schematic docking diagram of top five binding energy proteins. (**A**) PTGS2; (**B**) MAOB; (**C**) EGFR; (**D**) MAP2K1; and (**E**) PTSG1.

The average binding energy of the 37 targets was found to be −8.06 kcal/mol. The five targets with the lowest binding energy and their related metabolic pathways are shown in Table 4, and the docking position diagrams show the relative positions of the proteins and amino acid residues connected by hydrogen bonds (yellow dashed lines). The above results indicate that the targets obtained from bioinformatics analysis bind tightly to myricetin. The proteins with strong binding to myricetin were PTGS2, MAOB, EGFR, MAP2K1, and PTGS1 (Figure 7A–E).

### 3.8. RT-qPCR

**Figure 8:**
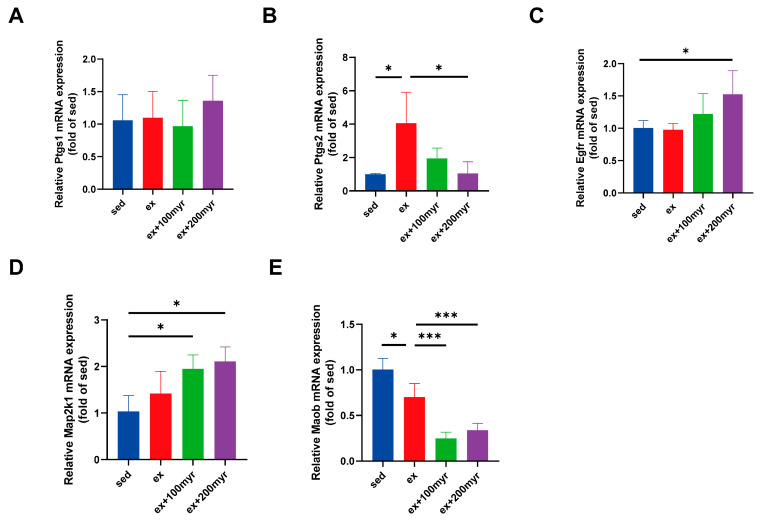
Transcriptional level of myricetin key targets. (**A**) Ptgs1; (**B**) Ptgs2; (**C**) Egfr; (**D**) Map2k1; and (**E**) Maob. The data are presented as mean ± SD. * *p* < 0.05; *** *p* < 0.001.

The results of RT-qPCR showed no significant changes in the transcriptional levels of Ptgs1 in all groups (Figure 8A). HIE significantly upregulated the transcriptional level of Ptgs2. Myricetin intervention significantly reduced the transcriptional level, which was more significantly decreased in the 200 myr group (Figure 8B). Myricetin increased the transcription level of Egfr, and the trends were more obvious in the 200 myr group (Figure 8C). Similarly, MAP2K1 transcript levels were significantly up-regulated by different concentrations of myricetin intervention (Figure 8D). The transcriptional levels of Maob were significantly decreased after intervention with different concentrations of myricetin (Figure 8E). In summary, the key targets obtained in the bioinformatics analysis showed significant changes in transcriptional levels for all protein targets, except Ptgs1.

## 4. Discussion

The adverse effects of HIE on the heart not only influence the health benefits of exercise but can also be detrimental to overall health and cause serious adverse consequences [20,21]. In this study, we explored the cardioprotective effects of myricetin in HIE in a mouse model. We found that myricetin intervention reduced the levels of serum myocardial injury markers, restored cardiac function, attenuated myocardial ultramicropathological changes, reduced the myocardial ischemic-hypoxic area, and increased CX43 levels.

As a plant compound, myricetin has a wide range of biological functions. The strategy of performing a combined analysis using network pharmacology and metabolomics contributed to a more comprehensive understanding of the metabolic regulatory mechanisms of myricetin [22]. The GO analysis of the predicted therapeutic targets of myricetin revealed that biological processes such as calcium channel regulation, lipid transport, phosphorylation of molecules, and G-protein-coupled receptors are closely related to myocardial activity [23,24,25,26]. The MCODE analysis of the PPI network identified hub biological processes dominated by protein phosphorylation and arachidonic acid metabolism. The regulation of protein phosphorylation is an important regulatory mechanism for the maintenance of myocardial function [27]. Arachidonic acid metabolism is associated with myocardial inflammation and multiple injury processes [28]. Metabolomics analysis revealed large changes in lipids and amino acids after 7 days of HIE, with significant changes in the expression of metabolites such as glutamate, glutamine, arachidonic acid, and myostatin, which are associated with myocardial injury [29]. The metabolic pathways with high enrichment were the FoxO pathway, glutamate metabolism, pentose phosphate pathway, and central carbon metabolism. The visualization of the commonly enriched pathways according to network pharmacology and metabolomics, as well as their included therapeutic targets and differential metabolites, demonstrated an extensive myocardial metabolic modulation and cardiac protection effect by myricetin [30,31,32].

Molecular docking and RT-qPCR revealed that the changes in Ptgs2, Maob, Egfr, and Map2k1 were consistent with bioinformatics predictions. Arachidonic acid metabolism, cellular lipid metabolism, central carbon metabolism, glycine-serine-threonine metabolism, histidine metabolism, and the FoxO metabolic pathway, where the above targets are located, may be the key pathways through which myricetin exerts its metabolic regulatory effects. PTGS2 is the key enzyme that converts prostaglandins to thromboxane and thus produces inflammatory damage [33]. Intervention with myricetin could significantly downregulate Ptgs2 transcriptional levels and thus inhibit the inflammatory response to exert a myocardial protective effect [34]. Ptgs1 is the key enzyme for the conversion of arachidonic acid to prostaglandin-like substances. Acting differently from Ptgs2, it mainly plays a protective role [35]. Although bioinformatic analyses and molecular docking suggest that myricetin may interact with Ptgs1, this is only a deduced theory. Through experimentation, it could be confirmed whether it actually changed [36], and RT-qPCR experiments confirmed that its transcript levels did not change. This suggests that myricetin exerts myocardial protective effects by inhibiting arachidonic acid-related validated metabolism rather than enhancing protective metabolism. MAOB is an isoform of monoamine oxidase that could worsen ischemia-reperfusion injury in cardiac myocytes [37]. In addition, monoamine oxidase is a key enzyme in glycine-serine-threonine and histidine metabolism, which can affect the synthesis of myostatin and the metabolism of myocardial energy substrates by regulating amino acid metabolism [38,39,40]. Myricetin could significantly downregulate the transcriptional levels of this gene and exert myocardial protective effects. Myricetin can also upregulate the expression levels of Egfr and Map2k1, which regulate the biological process of myocardial phosphorylation, producing a protective effect by reducing the myocardial inflammatory response and slowing myocardial fibrosis [41,42].

The beneficial effects of myricetin on the motor system have been reported previously. Wu et al. found that myricetin administration to mice altered skeletal muscle type and improved exercise performance by modulating the miR-499/Sox6 axis [43]. Zhang and his colleagues established a mouse model of myocardial injury through LPS intervention and found that myricetin reduced markers of myocardial injury and restored cardiac function, which may be related to the NF-κB signaling pathway [16]. Previous studies have searched for the mechanism of action of myricetin at the level of transcripts or proteins, and have achieved some results. However, they are unable to explain the complex pharmacological effects of flavonoid phytochemicals such as myricetin [44]. Our study focused on metabolism and used network pharmacology and metabolomics approaches to preliminarily elucidate the regulatory effect of myricetin in the metabolic network of the myocardium. Our study also provided new targets for metabolic intervention and research ideas for exercise protection and phytochemical research.

In summary, in this study, we explored the myocardial protective effects and metabolic mechanisms of myricetin in HIE by establishing an HIE model with intervention using different concentrations of myricetin. We found that intervention with myricetin could improve cardiac function, reduce myocardial injury markers levels, and alleviate pathological injury in HIE-group mice, and the mechanism might be mainly related to the myocardial metabolic network through the regulation of Ptgs2, Maob, Egfr, and Map2k1. This study provides a new direction for alleviating myocardial injury caused by HIE and a theoretical basis for the development of intervention programs. However, our research results were obtained from mice and future standardized human-related experiments are still needed to validate the cardioprotective effects of myricetin.

## Figures and Tables

**Table 1:** Databases used in the study.

Database	Function	Website
MetaboLights	Metabolomics data	https://www.ebi.ac.uk/metabolights (accessed on 30 January 2023)
SwissTarget prediction	Myricetin intervention target prediction	http://www.swisstargetprediction.ch (accessed on 3 August 2022)
String	Protein-protein interaction analysis	https://string-db.org (accessed on 10 August 2022)
Metascape	MCODE analysis	https://www.metascape.org (accessed on 10 August 2022)
Uniprot	Target protein crystal structure	https://www.uniprot.org (accessed on 25 August 2022)
PubChem	3D structure of myricetin	https://pubchem.ncbi.nlm.nih.gov (accessed on 25 August 2022)

**Table 2:** Software used in the study.

Software	Function	Version
R	GO and KEGG analyses and visualization	4.2.1
ImageJ	Image analysis	2.0.0
Cytoscape	Metabolic network visualization	3.9.1
Chem3D	Analysis of the 3D structure of myricetin	v20
PyMol	Removal of solvent molecules in protein molecules and visualization of molecular docking results	2.5.2
ADFRsuite	Converting file formats	1.0
AutoDock Vina	Molecular docking	1.1.2

**Table 3:** Primers used in RT-qPCR.

Gene	Forward (5′-3′)	Reverse (5′-3′)
Actb	GTGCTATGTTGCTCTAGACTTCG	ATGCCACAGGATTCCATACC
Ptgs1	ATGAGTCGAAGGAGTCTCTCG	GCACGGATAGTAACAACAGGGA
Ptgs2	TTCAACACACTCTATCACTGGC	AGAAGCGTTTGCGGTACTCAT
Egfr	GCCATCTGGGCCAAAGATACC	GTCTTCGCATGAATAGGCCAAT
Maob	ATGAGCAACAAAAGCGATGTGA	TCCTAATTGTGTAAGTCCTGCCT
Map2k1	AAGGTGGGGGAACTGAAGGAT	CGGATTGCGGGTTTGATCTC

**Table 4:** Top five binding energy proteins and metabolic pathways.

Protein	Binding Energy (kcal/mol)	Metabolic Pathway
PTGS2	−9.4	Arachidonic acid metabolism
		Regulation of lipolysis in adipocytes
MAOB	−9.3	Glycine, serine, and threonine metabolism
		Histidine metabolism
EGFR	−9.1	Central carbon metabolism in cancer
		FoxO signaling pathway
MAP2K1	−9.1	Central carbon metabolism in cancer
		FoxO signaling pathway
		cAMP signaling pathway
		Vascular smooth muscle contraction
PTGS1	−9.1	Regulation of lipolysis in adipocytes
		Platelet activation
		Arachidonic acid metabolism

## Data Availability

The original contributions presented in the study are included in the article/Supplementary Materials. Further inquiries can be directed to the corresponding author.

## Supplementary Materials

The following supporting information can be downloaded at: https://www.mdpi.com/article/10.3390/nu15061336/s1; Table S1: The ingredients of mice maintenance food; Table S2: The prediction of myricetin targets; Table S3: The relationship of enriched pathways between metabolomics and network pharmacology. Website of MTBLS5608: https://www.ebi.ac.uk/metabolights/MTBLS5608 (accessed on 1 March 2023).
